# Ictal and Interictal Cardiac Manifestations in Epilepsy. A Review of Their Relation With an Altered Central Control of Autonomic Functions and With the Risk of SUDEP

**DOI:** 10.3389/fneur.2021.642645

**Published:** 2021-03-12

**Authors:** Laure Mazzola, Sylvain Rheims

**Affiliations:** ^1^Neurology Department, University Hospital, Saint-Étienne, France; ^2^Lyon Neuroscience Research Center, INSERM U 1028, CNRS UMR, Lyon, France; ^3^Department of Functional Neurology and Epileptology, Hospices Civils de Lyon and University of Lyon, Lyon, France

**Keywords:** epilepsy, sudden unexpected death in epilepsy, heart rate variability, ictal asystole, ictal tachycardia and bradycardia

## Abstract

There is a complex interrelation between epilepsy and cardiac pathology, with both acute and long-term effects of seizures on the regulation of the cardiac rhythm and on the heart functioning. A specific issue is the potential relation between these cardiac manifestations and the risk of Sudden and Unexpected Death in Epilepsy (SUDEP), with unclear respective role of centrally-control ictal changes, long-term epilepsy-related dysregulation of the neurovegetative control and direct effects on the heart function. In the present review, we detailed available data about ictal cardiac changes, along with interictal cardiac manifestations associated with long-term functional and structural alterations of the heart. Pathophysiological mechanisms of these cardiac changes are discussed, with a specific focus on central mechanisms and the investigation of a possible deregulation of the central control of autonomic functions in addition to the role of catecholamine and hypoxemia on heart.

## Introduction

Since the description of the first ictal asystole more than 100 years ago (1), a large number of studies have investigated the complex inter-relationship between the brain and the heart in patients with epilepsy (2). Epilepsy-related cardiac manifestations can occur during seizures, but also in the inter-ictal period and can be associated with long-term functional and structural alterations of the heart. Over the past years, the scientific interest in these complex heart-brain interactions in patients with epilepsy have been reinforced by two main clinical reasons:

- The first corresponds to the issue of sudden unexpected death in patients with epilepsy (SUDEP). Among the causes of premature deaths in patients with epilepsy, SUDEP represents a major cause, especially in young adults with uncontrolled seizures with an incidence of about 0.5%/year of uncontrolled epilepsy (3). SUDEP is a non-traumatic and non-drowning death in patients with epilepsy, unrelated to a documented status epilepticus, in which postmortem examination does not reveal a toxicological or anatomic cause of death (3, 4). Although the exact pathophysiological mechanisms that lead to SUDEP remain unknown (5, 6), experimental and clinical data strongly suggest that most SUDEP result from a postictal central respiratory dysfunction progressing to terminal apnea, later followed by cardiac arrest (3). However, additional evidence suggests occurrence of an overall seizure-related failure of neuro-vegetative control (7), reinforcing the need of better understanding of the impact of seizures on cardiovascular function.
- The second aspect is the development of seizure detection devices, especially in order to alert the patients' caregivers and improve their safety. Because of the close relationship between seizures and changes in heart rate, cardiac monitoring has been proposed as a variable of choice for optimizing the detection rate of these devices (8).

Several reviews of the literature have analyzed the relationship between heart and epilepsy. Some have focused on ictal or interictal cardiac changes (9–12). Recently, Verrier et al. (13) proposed the concept of the “Epileptic Heart” as “a heart and coronary vasculature damaged by chronic epilepsy as a result of repeated surges in catecholamines and hypoxemia leading to electrical and mechanical dysfunction.” Others focused on new insights into possible pathways from epilepsy, catecholaminergic toxicity, subtle cardiac changes and sudden death (14), or on the implication of treatment (15).

In this review, the characteristics of ictal and interictal cardiac manifestations will be successively detailed. We will focus more particularly on their respective physiopathology, especially on central mechanisms with the investigation of a possible deregulation of the central control of autonomic functions, studied in functional imaging and using intracranial stimulations/recordings, in addition to the role of catecholamine and hypoxemia on heart which have already been reviewed elsewhere (13, 14). Their potential relations with SUDEP pathophysiology and implications in clinical practice, including for seizure detection, will be discussed.

## Characteristics of Seizure-Related Cardiac Manifestations

### Ictal Cardiac Manifestations

#### Heart Rate Changes

Tachycardia is the most common ictal cardiac manifestation. It is reported in 82% of patients on average, with some intra-individual variability, since not all seizures in a patient with ictal tachycardia will necessarily lead to tachycardia (9).

In the literature, changes in heart rate during seizures correspond on average to an increase of 30 bpm or more than 50% compared to the interictal heart rate. They mainly occur in the pre-ictal period or within 30 s after the beginning of the seizure, the maximal heart rate being achieved for a majority of seizures within the first 60 s (9, 16). However, most studies suggesting modifications of the heart rate in the pre-ictal period have been performed in patients investigated with scalp EEG, raising the possibility that concomitant ictal EEG discharge might have not been visible. In a study using intracranial electrodes, ictal tachycardia was always concomitant to an increase in unilateral ictal high frequency epileptic activity restricted to anterior hippocampus and amygdala (17). In addition, tachycardia is also frequently observed in the post-ictal period, particularly after tonic-clonic seizures (9, 18).

The percentage of seizures associated with a change in heart rate appears to be similar for generalized tonic-clonic seizures (64%) and for focal seizures (71%), although it is likely that the magnitude of the change is increased as the focal seizure progresses to bilateral tonic-clonic seizure (9, 19). In patients with focal epilepsies, tachycardia is more commonly seen during temporal lobe seizures than extra-temporal seizures (9, 20, 21). However, as most of studies have been performed in patients with temporal lobe epilepsy, a selection bias cannot be excluded. Although preferential right lateralization of seizures with tachycardia has been suggested, most studies do not find an association with the laterality of epileptic discharges (9, 20, 22).

Seizures with bradycardia or ictal asystole are much rarer. Ictal asystole, defined as a sinusal pause of at least 3 s occurring during a seizure, usually has a duration of <60 s, and is spontaneously reversible (23–27). They are only reported in focal seizures, and in 90% of cases they correspond to drug-resistant seizures with altered consciousness of temporal origin, without clear preferential lateralization. Incidence of ictal asystole in drug-resistant focal epilepsy is estimated at 12 per 100 patient-years (23–27). Distinguishing syncope related to ictal asystole from cardiac asystole might be difficult (28), and use of implantable loop recorder can sometimes be required in the diagnostic process. Older age at onset, occurrence during wakefulness, and brief duration of the events have been suggested to be in favor of cardiac asystole (29). Only rare patients with ictal asystole have undergone cardiopulmonary resuscitation (30), suggesting that the vast majority of seizures with asystole resolve spontaneously, without the need for resuscitation (18). However, the risk of recurrence is high (28). Unlike ictal tachycardia, ictal bradycardia, or asystole can be symptomatic with syncope and sometimes traumatic falls. Importantly, ictal bradycardia or asystole should be distinguished from post-ictal conduction or rhythm cardiac disorders. These complications, in particular severe bradycardia, asystole, or ventricular fibrillation, are closely related to post-ictal hypoxemia following central peri-ictal respiratory disorders (7, 31). After a generalized seizure, the risk of asystole is therefore greater in patients with severe post-ictal apnea (32). In the MORTEMUS study, which investigated respiratory and electrocardiogram (EKG) data from patients who died from SUDEP during long-term video-EEG, abnormal heart rhythms were observed after the onset of apnea in all deceased patients (33).

Other cardiac arrhythmias and conduction abnormalities, during or after seizures, have been reported in patients with drug-resistant focal epilepsy. Atrioventricular block, atrial fibrillation, supraventricular tachycardia, atrial, or ventricular premature depolarisations, ventricular fibrillation, and QT interval shortening or prolongation can thus be observed (9, 18).

#### Direct Cardiac Effects

Myocardial ischemia can be caused by seizures, especially in patients with cardiovascular risk factors. Up to 40% of seizures could be associated with ST segment depression (34). However, troponin remains normal in most patients (35, 36), suggesting that this transient myocardial ischemia does not generally result in severe acute ischemic myocardial injury. Seizures, especially generalized tonic-clonic seizures and status epilepticus, are also a well-known cause of Takotsubo syndrome, the clinical, EKG, and laboratory presentation of which may mimic that of acute ischemic heart disease (2). These complications have been linked to the release of catecholamines induced by seizures (36).

### Interictal Cardiac Manifestations

#### Changes in Myocardial Structure

It has been suggested both in experimental models and in patients, that recurrence of seizures can progressively lead to cardiac fibrosis, potentially through catecholaminergic toxicity (14). Compared to healthy matched controls, patients with temporal lobe epilepsy have higher left ventricular rigidity, linked to cardiac fibrosis by deposits in the extracellular matrix, which in turn promotes systolic and diastolic dysfunction and arrhythmogenesis (37). Although the relationship between these long-term structural changes and the risk of ictal arrhythmias remains to be determined, several studies have reported an association between cardiac fibrosis and the risk of SUDEP (38, 39).

#### Channelopathies

More recently, it has also been shown that repetition of seizures can alter the expression of cardiac ion channels. Epilepsy-related alterations in the cardiac expression of sodium (Nav1.1/1.5), potassium (Kv4.2/4.3), calcium (NCX1), and cationic (HCN2/4) channels have thus been reported in animal models (40). It remains to be determined whether or not this mechanism is associated with impaired vegetative regulation in patients with epilepsy and especially, with the risk of SUDEP.

#### Heart Rate Variability (HRV)

HRV is the change in the time interval between two heart beats. HRV reflects the balance between sympathetic and parasympathetic activity of the autonomic nervous system. HRV is thus an index of activity of the neurovegetative system, whose decrease is a strong predictor of sudden death in patients with heart disease (41). Overall, increased heart rate variability indicates a shift toward parasympathetic dominance, while lower heart rate variability is seen in times of high sympathetic output (42). In patients with epilepsy, HRV is usually decreased, suggesting a shift toward sympathetic dominance (42). This has been shown in various types of epilepsy, including temporal lobe epilepsy (43, 44), frontal lobe epilepsy (45), idiopathic generalized epilepsy (44), epileptic spasms (46), or in Dravet Syndrome, where patients have extremely depressed parasympathetic function (10, 47), even in comparison with other types of epilepsy. In addition, it has been suggested that alteration of HRV might be precipitated and/or aggravated by insular resection in patients undergoing epilepsy surgery (48). However, the exact relationship between these chronic alterations of HRV and the risk of SUDEP remains unclear (7). Some studies reported association between risk of SUDEP and severe alteration of HRV (10, 49–51) whereas others did not confirm this observation (52). Furthermore, the alterations of HRV might also be associated with other risk SUDEP factors, including post-ictal generalized EEG suppression (53).

In addition, many studies have examined peri-ictal changes in HRV (10, 54). The results are sometimes heterogeneous, but overall seem to show an increase in sympathetic activity during the seizure, regardless of the type of seizure, but more markedly for temporal seizures and generalized seizures (7). Recovery occurs gradually, as post-ictal changes that can be prolonged, up to several hours. Changes in HRV can precede clinical onset of seizure by several seconds and have therefore been studied for the development of seizure detection tools.

## Pathophysiological Mechanisms

The mechanisms underlying the emergence of these cardiac alterations remain poorly understood and several hypotheses need to be considered. These hypotheses may coexist in the same patients, interacting with each other.

### Deregulation of Central Control of Vegetative Functions

**Figure 1:**
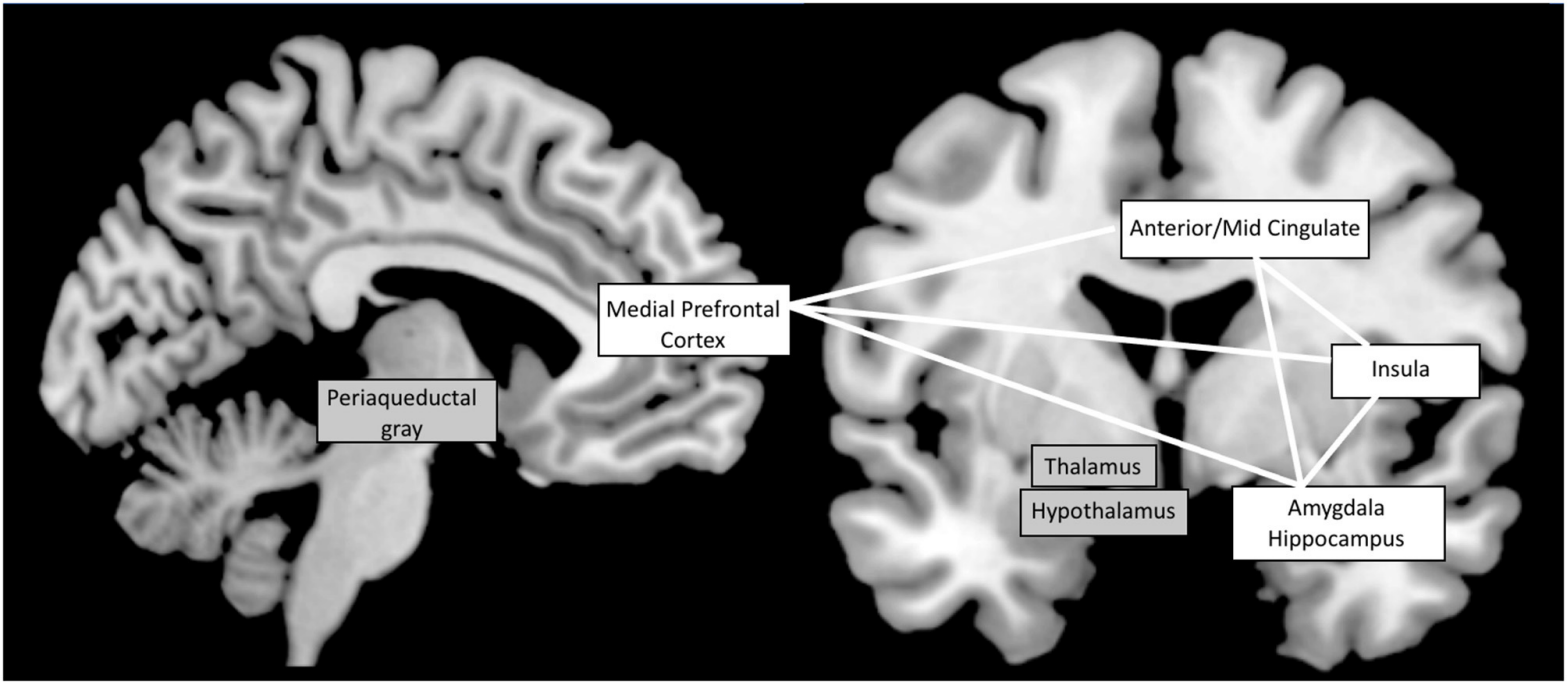
The Central Autonomic Network: most implicated brain regions according to functional neuro-imaging studies (57, 60, 62). Cortical structures are shown on a white background while subcortical structures are on a gray background. White lines symbolize the functional connectivity between cortical regions.

Some of the most important integrative control centers for autonomic nervous system functions are located in the brainstem (55, 56). However, many animal and human studies support that cortical regions are involved in autonomic function and modulation in response to environment changes (55, 57–60). In 1993, Benarroch proposed the term of “Central Autonomic Network” (CAN) to describe a group of forebrain, brainstem, and limbic regions involved in the generation of an appropriate autonomic functional state (55). In 2000, Thayer and Lane (61) proposed a model of neurovisceral integration, permitting to link cardiac regulation to emotional or cognitive tasks through activation of the CAN. In addition to the autonomic nuclei of the brainstem and limbic structures such as the amygdala and the insula, their model also includes the cingulate and medial prefrontal cortex. Cortical regions, particularly medial prefrontal cortex, would exert a top-down control on cingulate, anterior insula and amygdala, which form an interconnected network, and modulate activity of subcortical and brainstem regions. These later regions would in turn finalize the autonomic output to the body by modulating the parasympathetic/sympathetic balance. In accordance with this model, recent meta-analysis of human neuroimaging experiments evaluating central autonomic cardiovascular processing identified several consistently implicated brain regions, consisting of cortical areas, including the anterior and mid-cingulate cortices, insula, amygdala, hippocampus, medial prefrontal cortex; and subcortical structures such as thalamus, hypothalamus, periaqueductal gray matter (57, 60, 62) (see Figure 1). Orbitofrontal cortex is also mentioned by some authors (60, 62). Analysis of functional connectivity has revealed functional connectivity between the medial prefrontal cortex and other structures of the CAN, particularly the amygdala and the hippocampus (63, 64). Parcellation of orbitofrontal cortex and hypothalamus has shown specificity for functional connectivity between the medial orbito-frontal cortex and the medial hypothalamus (65, 66). In their meta-analysis, Thayer et al. (66), established a link between mainly amygdala and ventromedial prefrontal cortex activation, during several cognitive and affective tasks, and heart rate variation. De la Cruz et al. (67) recently investigated the relationship between heart rate and functional connectivity of brain regions involved in autonomic control. Subjects with slow heart rate exhibited significantly increased functional connectivity between amygdala, insula, prefrontal cortex, anterior cingulate, hippocampus, and hypothalamus compared to subjects with medium or fast heart rate.

Some studies suggested the possibility of a lateralization of insular cortex in terms of cardiac function, the right insula being more involved in sympathetic regulation, and the left one in parasympathetic cardiac regulation [see (60, 68) for a review]. On the contrary, others did not conclude to any lateralization (57).

Very few studies have investigated the effects of cortical stimulation on heart rate in humans. Electrical stimulation of limbic structures, especially the amygdala and peri-amygdaloid pyriform cortex, have been reported to produce autonomic changes, including cardiovascular responses, mediated by either sympathetic or parasympathetic pathways (69). Autonomic responses (including heart rate changes) are also mentioned after stimulation of the cingulate cortex (70, 71) and orbito-frontal cortex (72). Several data support the pivotal role of the insula in this central autonomic network, sometimes with contradictory experimental results. In animals, stimulation of rat posterior insula (58, 73) and primate antero-ventral insula (74, 75) have induced heart rate changes. In humans, Oppenheimer et al. (59) were the first to report heart rate changes after 70 intraoperative stimulations of the insula in five patients. Bradycardia was observed more frequently after stimulations of the left insula, whereas tachycardia was more often elicited after stimulation of the right insular cortex. More recently, Chouchou et al. (76) confirmed the role of insula in regulation of cardiac activity, based on responses to direct electrical stimulation performed during stereo-electro-encephalography. Out of 100 insular stimulations, almost 50% induced a modification of heart rate. Insular representation of tachycardia was more posterior than that of bradycardia and both types of cardiac responses were equally represented in right and left insula. Tachycardiac responses were underpinned by sympathetic reactivity, and bradycardia by parasympathetic control.

Likewise, Catenoix et al. (77) reported an insular seizure with ictal asystole. The electrode implanted in the left posterior long gyrus showed a high frequency discharge starting 2 s before asystole, underlying the possibility of a proper role of insula in some dysautonomic seizures. However, a recent SEEG study exploring 37 temporal lobe seizures in 9 patients, showed that tachycardia was concomitant to an increase in epileptic activity in anterior hippocampus and amygdala, but was independent of ictal insular activity (17), suggesting that insula implication is not necessary to evoke cardiac changes.

Sympathetic or parasympathetic ictal changes could so result from direct activation by epileptic discharge of the central autonomic network (78), whose activation by the discharge would modify the activity of the autonomic nervous system during the seizure. In addition, like the data which show a progressive alteration of the brainstem structures involved in respiratory control (79), it could be possible that the repetition of the seizures could modify the subcortical nuclei in charge of vegetative regulation. Thus, the repetitive stimulation of central autonomic network by epileptic discharges may lead to chronic dysfunction of the autonomic nervous system leading at least in part to interictal disorders.

### Genetic Background

Several ion channel genes whose mutations are involved in cardiac arrhythmias are also expressed in the brain. For example, the SCN5A gene, whose mutation is associated with long QT syndrome, is also expressed in the brain and is associated with epilepsy (80). Some cardiac events, including sudden deaths, may therefore be linked to genetic risk factors common to epilepsy and cardiac arrhythmias.

A growing body of evidence points to a genetic susceptibility to cardiorespiratory and autonomic dysfunction in epilepsy. In an analysis of the entire exome sequencing of 61 SUDEP cases, mutations known to cause long QT syndrome were found in 7% of cases and an additional 15% had candidate variants in potentially predisposing genes to malignant cardiac arrhythmias (81). Similarly, the effect of the SCN1A mutation on heart function may partly explain the increased risk of mortality in Dravet syndrome (82–84).

### Roles of Epilepsy Treatments

Several anti-seizure drugs have been associated with conduction abnormalities or arrhythmias. This has been particularly reported with sodium channel blockers (2), including risk of atrioventricular block with carbamazepine (85), sinus pause and hypotension with rapid administration of phenytoin (86) or atrioventricular block or atrial fibrillation with lacosamide (87–89). However, no formal relationship has been established between these drug-related adverse events and ictal arrhythmias (2). Importantly, a pooled analysis did not find a significant association between the treatments and an increased risk of SUDEP when adjusting the frequency of generalized tonic-clonic seizures (90).

The effect of vagus nerve stimulation (VNS) on autonomic function remains uncertain. Heart changes associated with VNS are rare. Few cases of VNS-induced bradycardia have been reported. In addition, data on the alterations in parasympathetic tone of the cardiovascular system induced by VNS are contradictory (91).

Finally, while the data concerning the relationship between some antiepileptic treatments, in particular enzyme inducers, and the destabilization of lipid metabolism are numerous (92), the real impact of these modulations on the risk of atherosclerosis and a fortiori on the risk of the occurrence of cardiovascular events remain debated (93–95). In a study based on an insurance registry from several states in the United States, the risk of having a stroke with enzyme inducer antiepileptics compared to other treatments was 1.22 (0.90–1.65) (94). A large British study used the GPRD database and studied 252,407 patients over the age of 18 who received antiepileptic therapy between 1990 and 2013 (95). Among them, 5,069 strokes (ischemic or hemorrhagic) and 3,636 myocardial infarctions have been reported. The use of enzyme-inducing therapy was not associated with a significant increase in the risk of stroke, including ischemic stroke. In contrast, the use of an inducer for more than 24 months was associated with a significantly increased risk of myocardial infarction [1.46 (1.15–1.85)] (95). Nevertheless, translated into annual risk, this difference remained very low, with a difference in risk of occurrence of 1.39 / 1,000 patients per year (0.33–2.45).

## Potential Relations With Pathophysiology of SUDEP

Epilepsy-related cardiac dysfunction may be associated with increased risk of premature mortality, because of a relation either with the risk of SUDEP or with the risk of heart diseases. As discussed in details by Verrier et al. the issue of long-term risk of heart diseases might be predominant in terms of incidence and should deserve a specific attention (13). This risk might be primarily related to the direct effects of seizures on the heart, the genetic background and/or the long-term adverse events of antiseizure drugs (13). In contrast, the exact relation between these cardiac symptoms and the risk of SUDEP remains to be clarified.

The data suggesting that the ictal cardiac dysfunction plays a key leading role in the initiation of the cascade of events that lead to SUDEP are limited. As discussed above, the possibility that the main event is a serious heart rhythm disorder seems unlikely or may represent a minority of SUDEP (5, 27). Although severe bradycardia, transient asystole or an episode of ventricular tachycardia was observed in all monitored SUDEP in the MORTEMUS study, these events followed chronologically the apnea (33). Even in Dravet syndrome, in which it is possible that the channelopathy also has a cardiac effect, available data are conflicting. In some rodent model of Dravet Syndrome, altered cardiac electrical function contributed to susceptibility to arrhythmogenesis and SUDEP (82). However, in others, asystole was shown to be also triggered by postictal respiratory dysfunction, possibly by a direct effect of hypoxemia on heart muscle (96).

Accordingly, an important aspect might be the interrelations between the central regulation of respiratory function and the one of neurovegetative functions, including the central regulation of cardiac rhythm. Brain areas involved in these regulations are highly connected to each other, both at the cortical level and in the brainstem, and each of them is partly regulated by the other. Brain regions involved in the regulation of the arterial pressure as well as in breathing control thus overlap with the Central Autonomic Network involved in the regulation of heart rhythm, both at the cortical level and in the brainstem. Direct electrical cortical stimulation of the subcallosal neocortex resulted in consistent decreases in systolic blood pressure (97). The latter was interpreted as a reduction in sympathetic drive, resulting in a reduction in cardiac output (97). Similarly, direct electrical cortical stimulation of several areas of the Central Autonomic Network reliably induces apnea. This has mostly been reported in the amygdala or the hippocampus (98–100) but also in the cingulate and orbitofrontal cortex (101). In addition direct electrical stimulation of the perisylvian cortex can result in significant decrease of SpO_2_ (102). In this context, it might be speculated that acute disorganization of these cortical regions by an epileptic discharge might precipitate simultaneous alterations of the cortical drive of respiration, cardiac rhythm, and arterial pressure. Some clues obtained during seizures might be in favor of this hypothesis. It has thus been shown that ictal autonomic dysfunction is correlated with seizure-related respiratory dysfunction in temporal lobe seizures, with prolonged impairment of parasympathetic tone associated with postictal hypoxemia (54). In generalized convulsive seizures, there is a close relationship between post-ictal severe respiratory dysfunction and post-ictal conduction or rhythm cardiac disorders (32, 33). In this seizure type, which is the main risk factor of SUDEP (103), the cortical dysfunction of neurovegetative regulation and breathing control, might be reinforced by the dysfunction of brainstem control, resulting from the spreading depolarization in the brainstem. In a rodent model of SUDEP, pharmacological-induction in the brainstem of electroencephalographic suppression resulted in apnea, bradycardia, and asystole, similar to the events seen in monitored SUDEP (104). Furthermore, respiratory regulation following a seizure is modulated by norepinephrine pathway (105). In patients, the occurrence and/or severity of post-ictal EEG suppression is associated with post-ictal respiratory dysfunction (106) as well as with both sympathetic activation and parasympathetic suppression (53).

Despite these preliminary data, the hypothesis of a leading role of post-ictal central neurovegetative breakdown in the SUDEP requires additional evidence. In addition, the exact relationship between these potential peri-ictal alterations of the Central Autonomic Network, long-term alterations of respiration and long-term alteration of cardiac regulation, especially HRV, remains an open question. Whether or not the risk of severe of post-ictal neurovegetative breakdown, and eventually SUDEP, might be higher in patients with combined alterations of respiratory and cardiac controls is unknown. Better understanding how these issues interact with each other and if they share pathophysiological mechanisms might be of key importance for unraveling SUDEP biomarker with greater predictive value than those currently available (7), a critical aspect in the perspective of SUDEP prevention (107).

## Implications in Clinical Practice

### Diagnostic and Management of Epilepsy-Related Cardiac Disorders

Identification of interictal cardiac changes should allow the prevention, early detection, and possible treatment of cardiac co-morbidities. It could also guide the choice of anti-seizures drugs according to the patient profile, in order to avoid the appearance or worsening of arrhythmia or cardiac conduction disorders. An EKG should therefore be performed in all patient with newly-diagnosed epilepsy, especially to exclude long-QT syndrome, but it should then be reprocessed regularly in the follow up. Some studies have suggested the interest of prolonged routine EKG recordings (108).

Similarly, identification of patients with severe ictal heart rate changes is important. Although ictal asystole are typically self-limiting events, they can expose to severe injuries. Considering the risk of seizure-related traumatisms and the risk of recurrence, aggressive treatment, including pacemaker implantation, should be discussed if seizure freedom cannot be achieved (28). Because active management of antiseizure drugs might reduce the risk of SUDEP (109), whether or not identification of post-ictal cardiac arrythmias after generalized convulsive seizure (32) should be taken into account in therapeutic decision is an open question.

### Seizure Detection Devices

Over the past 10 years, there has been a growing interest in the potential applications of mobile health technologies for seizure detection, with the objective of faster caregivers' intervention and decreased risk of seizure-related injuries. Basically, three physiological variables can be used for non-EEG based seizure detection: detection of body movements, eye movements, and seizure-related modification of vegetative functions, including the cardiac rhythm (110). While detection of generalized tonic-clonic seizures has shown promising results with utilization either alone or in combination of accelerometers, automatic video detection, surface EMG, and bed alarms (8, 111), these approaches are much less sensitive for focal seizures. In contrast, the main approach consists in detection of seizure-induced autonomic changes, especially cardiac rhythm changes. While first studies showed disappointing results with high rate of false-alarm, recent data were more encouraging. In a recent study using a wearable EKG device, the overall sensitivity was low at 54% but raised to 90.5% for non-convulsive seizures in the 53.5% of patients in whom more than 66% of seizures were detected (112). An ictal change in HR of more than 50 bpm (increase or decrease) predicted responders with a predictive positive value of 87% (95% CI 69.9–95.4%) and a negative predictive value of 90% (95% CI 70.4–97.2%) (112).

Beyond detecting ictal tachycardia to alert caregivers about the occurrence of a seizure, an additional question will be how these devices can be used to detect post-ictal arrythmia and/or asystole. Such approach might be used to alert patients family or the rescue services of a severe post-ictal arrythmia with high risk of immediate SUDEP, especially in patients with frequent nocturnal convulsive seizures and who sleep alone (113).

## Conclusion

Much progress has been made in recent years in the characterization of ictal and interictal cardiac manifestations in epilepsy. Although their pathophysiology remains debated, improving knowledge could lead us to improve the care of our patients. Their identification should allow the prevention and possible treatment of cardiac co-morbidities, and also guide the choice of anti-epileptic treatments, in order to prevent the appearance or worsening of conduction or rhythm cardiac disorders. In addition, monitoring EKG and HRV, which are biomarkers easy to record and measure, could allow the development of increasingly precise non-invasive seizure detection tools for monitoring and possibly for the early treatment of seizures. However, a key remains to better understand the exact relation between these cardiac manifestations and the risk of SUDEP. Further studies are required to decipher the respective role of centrally-control ictal changes, long-term dysregulation and direct effects on the heart function.

## Author Contributions

All authors listed have made a substantial, direct and intellectual contribution to the work, and approved it for publication.

## Conflict of Interest

SR received consultant and/or speaker fees from EISAI, UCB Pharma, Livanova, GW Pharma, Arvelles therapeutics, Idiorsia. The remaining author declares that the research was conducted in the absence of any commercial or financial relationships that could be construed as a potential conflict of interest.
